# Bioequivalence studies in Morocco

**DOI:** 10.11604/pamj.2020.36.279.22241

**Published:** 2020-08-14

**Authors:** Sanaa Zaoui, Wafaa Fadili

**Affiliations:** 1 Department of Pharmacology and Toxicology, University Hospital Center Mohammed VI, Marrakech, Morocco,; 2 Department of Nephrology, University Hospital Center Mohammed VI, Marrakech, Morocco

**Keywords:** Generic drugs, bioequivalence, Morocco, legislative texts, clinical trials

## To the editors of the Pan African Medical Journal

The development of the generic drug represents one of the strongest axes of the regulation of drug expenditure. In addition to saving the cost of the medical treatment that it allows, it has other interests. Among other things, the fact that it is among the best tools that will allow the viability and success of basic medical coverage in our country [1]. The law mandated bioequivalence trials in 2006. A decree of the Ministry of Health, effective in June 2012, strengthened the law, recalling the legal obligation to demonstrate the bioequivalence of a generic drug with its brand-name drug, before granting marketing authorization for any generic drug manufactured locally or imported. It established the cases where bioequivalence studies are required, the cases justifying the dispensation of bioequivalence studies and provided a precise definition of the notion of bioequivalence, bioavailability and that of the brand-name drug [2]. Since then, the first bioequivalence center authorized by the Ministry of Health has been created in September 2015. Pharmaceutical manufactures have so far been studying bioequivalence abroad, particularly in Jordan, France, Egypt and India. This center will allow Moroccan industrialists to follow directly the progress of the studies and a cost saving, this one is lower of 20% than abroad. It will also allow them to benefit from a service offering quality and proximity [3]

To modify and supplement the 2012 decree, which has experienced some shortcomings and limitations noted during its implementation, but also to comply with the latest recommendations of the World Health Organization (WHO), and new international standards in this regard, the Ministry of Health published a new decree on bioequivalence in March 2019. This decree underlines the obligation for any pharmaceutical industry, marketing generic drugs to file a bioequivalence study, when submitting the application for renewal of the marketing authorization for these medicines. This project also sets the necessary conditions for the realization of the bioequivalence studies and the scientific criteria justifying the exemption from these studies [4]. This legal text, which will strengthen the legislative and regulatory provisions for the generic drug, supports the national policy on medicines and health products, through the provision of quality medicines, effective and at reasonable prices taking into account the purchasing power of citizens. Probably with these legal texts, there will gradually be an increase in the number of bioequivalence studies done in Morocco and their generalization to cover all generic drugs requiring these studies, especially that they exist voices that ask the industrialists to prove the bioequivalence of their generics and the publication of the results [5]. In addition to legal texts, and to meet increasing demand, other bioequivalence centers must be created and authorized.
